# Genetic alterations of Cyclin D-CDK4/6-INK4-RB pathway in prostate cancer

**DOI:** 10.1007/s11033-025-10531-1

**Published:** 2025-04-30

**Authors:** Monika Kmeťová Sivoňová, Márk Híveš, Ján Kliment, Róbert Dušenka, Marián Grendár, Daniel Evin, Peter Kaplán, Martina Knoško Brožová, Marta Vilčková, Andrej Vondrák, Jana Jurečeková

**Affiliations:** 1 Department of Medical Biochemistry, Jessenius Faculty of Medicine in Martin, Comenius University in Bratislava, Martin, Slovak Republic; 2 Department of Urology, Jessenius Faculty of Medicine in Martin and University Hospital Martin, Comenius University in Bratislava, Martin, Slovak Republic; 3 Biomedical Center Martin, Jessenius Faculty of Medicine in Martin, Comenius University in Bratislava, Martin, Slovak Republic; 4 Department of Nuclear Medicine, Jessenius Faculty of Medicine in Martin and University Hospital Martin, Comenius University in Bratislava, Martin, Slovak Republic; 5 Izotopcentrum, s.r.o, Nuclear Medicine Department, Nitra, Slovak Republic; 6 Department of Medical Biochemistry, Jessenius Faculty of Medicine in Martin, Comenius University in Bratislava, Malá Hora 4D, Martin, 03601 Slovakia

**Keywords:** Cell cycle, Polymorphism, Expression, Prostate cancer, Slovak population

## Abstract

**Background:**

Alterations in key cell cycle regulators are strongly linked to tumorigenesis. Therefore, we hypothesized that polymorphisms of genes encoded cyclin-dependent kinase 4 and 6 (CDK4 and CDK6), cyclin D1 (CCND1), CDK inhibitors p16^INK4a^ and p15^INK4b^, as well as the retinoblastoma protein (RB), could modulate prostate cancer risk and influence the corresponding mRNA levels.

**Methods and results:**

We evaluated *CDK4* rs2069502, *CDK6* rs2285332, *CCND1* rs9344, *p16*^*INK4a*^ rs11515, *p15*^*INK4b*^ rs3217986, and *RB* rs3092904 polymorphisms using TaqMan^®^ SNP Assays in a cohort comprising 532 prostate cancer patients and 567 control subjects. Additionally, we measured the relative mRNA expression levels of genes encoding these proteins in RNA derived from 44 prostate tumor tissues and 31 benign prostatic hyperplasia (BPH) tissues using quantitative real-time PCR (qRT-PCR). No statistically significant associations were found between the *CDK4* rs2069502, *p16*^*INK4a*^ rs11515 and *RB* rs3092904 polymorphisms and prostate cancer risk. However, the *GA* genotype of *CCND1* rs9344 polymorphism was significantly associated with an increased risk of prostate cancer (OR, 1.64; 95% CI, 1.23–2.20; *p* < 0.001). Moreover, the relative mRNA expression levels of *CCND1*, *p15*^*INK4b*^ and *RB* were significantly lower (*p*<0.05) in prostate tumor tissues compared to BPH tissues. Furthermore, lower relative expression levels of *CDK4* and *p16*^*INK4a*^ mRNA were associated with elevated serum PSA levels (≥10 ng/ml; *p*<0.05), while reduced relative expression of *p15*^*INK4b*^ was correlated with a higher pathological T stage (pT3/pT4; *p*<0.05).

**Conclusions:**

Our findings indicate that genetic alterations, including polymorphisms and/or gene expression changes in the cyclin D1-CDK4-p16^INK4a^/p15^INK4b^-RB pathway, are associated with prostate cancer risk.

## Introduction

Prostate cancer is a highly heterogenous malignancy that predominantly affects elderly males [1]. The risk, aggressiveness, and prognosis of prostate cancer vary substantially based on race, ethnicity, and geographic location. Its incidence is highest in more developed regions such as North America, and Western and Northern Europe, and lowest in areas like East, Southeast, and Southcentral Asia. This variation might be partially explained by differences in early population-based screening practices such as prostate-specific antigen (PSA) testing [2]. Well-established risk factors for prostate cancer include age, ethnicity, and family history, as well as exogenous factors such as diet, smoking, physical activity and environmental factors [3]. The treatment of prostate cancer often involves various therapeutic approaches, either individually or in combination, comprising androgen-deprivation therapy, radiation therapy, ablative therapies, chemotherapy, and immunotherapy [4]. Emerging evidence underscores the importance of identifying appropriate molecular therapeutic targets as a critical step toward advancing precision oncology in the management of prostate cancer.

Dysregulation of the cell cycle is a hallmark of cancer. Progression through each phase of the cell cycle is regulated by checkpoints that enable cells to respond to DNA damage, either by halting the cell cycle or inducing apoptosis [5–7]. Upon activation by mitogenic signals, D-type cyclins bind to and activate CDK4 and CDK6 [8]. The activated cyclin D-CDK4/6 complex phosphorylates the RB leading to its dissociation from the E2F transcription factors. This initiates the transcription of genes required for cell cycle progression, including those encoding E-type cyclins (cyclins E1 and E2). Cyclin E subsequently binds to CDK2, forming the cyclin E-CDK2 complex, which completes RB phosphorylation during the G_1_ phase. Hyperphosphorylated RB releases E2F transcriptional activity, driving the expression of genes required for initiation of DNA synthesis and entry into the S phase [8, 9]. The activity of the cyclin D-CDK4/6 complex is inhibited by p16^INK4a^ (encoded by the *CDKN2A* gene) and p15^INK4b^ (encoded by the *CDKN2B* gene), both of which are members of the INK4 family of cell cycle inhibitors. These proteins contribute to G_1_ arrest by directly binding to CDK4 or CDK6, thereby inhibiting their catalytic activity [7].

Gene polymorphisms or variations in the expression of key cell cycle regulator genes may disrupt the normal regulation of cell growth and division, contributing to cancer development [10]. Previous studies have revealed an association between the *CCND1* rs9344 polymorphism and an increased risk of prostate cancer [11–13]. However, the potential associations between other polymorphisms – such as *CDK4* rs2069502, *CDK6* rs2285332, *p16*^*INK4a*^ rs11515, *p15*^*INK4b*^ rs3217986, *RB* rs3092904 – and prostate cancer risk have not yet been elucidated.

In this study, we aimed to estimate the contribution of these selected polymorphisms (*CDK4* rs2069502, *CDK6* rs2285332, *CCND1* rs9344, *p16*^*INK4a*^ rs11515, *p15*^*INK4b*^ rs3217986, *RB* rs3092904) within the cyclin D-CDK4/6-INK4-RB pathway to prostate cancer risk. Additionally, we examined whether these selected polymorphisms were associated with differential expression levels of the corresponding genes (*CDK4*,* CDK6*,* CCND1*,* p16*^*INK4a*^, *p15*^*INK4b*^, and *RB*).

## Materials and methods

### Study population

The study cohort comprised of 1099 men, comprising 532 prostate cancer patients and 567 healthy controls, who were recruited at the Department of Urology of University Hospital in Martin and the Jessenius Faculty of Medicine in Martin, Comenius University, between May 2005 and December 2020. Most of the participants were also enrolled in our previous studies [14, 15]. The prostate cancer patients (*n* = 532) had undergone either radical prostatectomy, or transurethral resection of the prostate, or prostatic biopsy. Within the entire cohort, 230 (43%) of 532 patients underwent the radical prostatectomy as the primary treatment. Of the rest of the patient’s cohort, 300 (57%) of 532 cases had undergone transurethral resection of the prostate or prostatic biopsy. Case status was confirmed by medical records, including the clinical and pathologic tumor-node-metastasis (TNM) stage and the Gleason scores at biopsy and at radical prostatectomy.

All participants, including prostate cancer patients, healthy controls, and patients with BPH, were Caucasian ethnicity. Informed consent was obtained from all participants, and the study was approved by the Ethical Boards of the Jessenius Faculty of Medicine in Martin, Comenius University. The number of subjects in each group, the median ages of prostate cancer patients at the time of diagnosis and of controls at the time of interview and clinicopathological characteristics (serum PSA levels, Gleason score and pathological T stage), are summarized in Table 1. Serum PSA levels were measured in cases, controls and patients with BPH using the Beckman Coulter Access^®^ Hybritech^®^ assay (Beckman Coulter, Inc., Brea, California, USA) following the manufacturer’s protocol. All control participants were cancer-free and had normal serum PSA levels. Blood samples (3 ml) were collected from all participants in EDTA vials for further analysis.

**Table 1:** Characteristics of the study groups

	Healthy controls	Prostate cancer
**Number**	567	532
**Age (years)**		
Median (IQR)	65 (58–72)	67 (61–73)
**PSA (ng/ml)**		
Median (IQR)	2 (1–5)	**10** (6–23)
**Gleason score**		
≤7	NA	283 (53.2%)
> 7	NA	148 (27.8%)
Missing	NA	101 (19.0%)
**Pathological stage**		
pT1/pT2	NA	117 (22.0%)
pT3/pT4	NA	113 (21.0%)
Missing	NA	302 (57.0%)

IQR: Interquartile range. NA: Not applicable. Bold characters represent *p* < 0.05

Forty-four radical prostatectomy specimens and 31 BPH specimens were analyzed from patients undergoing surgery at the Department of Urology, University Hospital in Martin, and the Jessenius Faculty of Medicine in Martin, Comenius University. Pathological evaluation of these tissues was confirmed. Thirty-one patients underwent transurethral resection of the prostate for symptomatic BPH. Histologic review of the prostate tissues ensured the absence of prostate cancer in these 31 samples. The median age of patients in the prostate cancer and BPH cohorts was 71.5 years (range: 55–88) and 73 years (range: 58–82), respectively. Total serum PSA levels measured at the time of diagnosis were significantly higher among prostate cancer cases (median: 12.5 ng/ml, range: 2.2–161.0 ng/ml) compared to BPH patients (median: 4.37 ng/ml, range: 0.7–22.0 ng/ml; *p* < 0.01). Tumor tissue specimens were categorized based on Gleason score (≤ 7: 33%; >7: 67%) and pathological tumor stage (pT1/pT2: 36%; pT3/pT4: 64%). All tissue samples were immediately preserved in an mRNA-stabilizing solution (RNAlater^®^; Applied Biosystems/Ambion, Waltham, MA, USA) and stored at -80 °C until further analysis.

### DNA extraction and genotyping

Genomic DNA was extracted from peripheral blood samples using the Wizard^®^ Genomic DNA Purification Kit (Promega, Madison, WI, USA) and ethanol precipitation according to the manufacturer’s protocol. The extracted genomic DNA was stored at -20 °C until further processing.

*CDK4* rs2069502, *CDK6* rs2285332, *CCND1* rs9344, *p16*^*INK4a*^ rs11515, *p15*^*INK4b*^ rs3217986 and *RB* rs3092904 polymorphisms were genotyped using TaqMan^®^ SNP Assays (Thermo Fisher Scientific, Waltham, MA, USA), as described previously [15]. Briefly, the allelic specificity of the TaqMan^®^ SNP assay was provided by two fluorogenic probes, one labelled with FAM dye and the other with VIC dye. TaqMan^®^ SNP genotyping assays were supplied at 40× concentration. The master mix for PCR contained 0.25 µl of TaqMan^®^ SNP genotyping assays mix (20×; prepared by diluting the 40× stock solution with 1× TE buffer), 5 µl of TaqMan^®^ Genotyping Master Mix (2× Thermo Fisher Scientific), and 4.5 µl of genomic DNA at a concentration of 10 ng. PCR amplification was performed on a Viia7 Real-Time PCR System (Thermo Fisher Scientific) according to the manufacturer’s protocol with the following thermal cycle conditions: one cycle at 60 °C for 30 s, one cycle at 95 °C for 3 min, 40 cycles of 15 s at 95 °C, and 1 min at 60 °C, followed by a post-read step at 60 °C for 30 s. Genotype calls were obtained by analyzing the data using the X-Y scatter plot in Viia7 software (Thermo Fisher Scientific).

### qRT-PCR assay

Total RNA was extracted with the AllPrep DNA/RNA/miRNA Universal Kit (Qiagen GmbH, Hilden, Germany) following the manufacturer’s instructions. The RNA concentration and purity were assessed spectrophotometrically using a Nanophotometer (Implen GmbH, München, Germany). cDNA was subsequently synthesized from the extracted RNAs using RT^2^ First Strand Kit (Qiagen GmbH) in accordance with the manufacturer’s protocol.

Quantitative PCR (qPCR) was performed on a Viia7 Real Time PCR System (Thermo Fisher Scientific) and a custom RT^2^ Profiler PCR array (Qiagen GmbH) was used as describes previously [15]. Gene expression data were analyzed using the ΔΔCt method, normalizing to the average expression levels of the housekeeping genes glyceraldehyde-3-phosphate dehydrogenase (GAPDH) and β-actin in each sample.

### Statistical analysis

Data were explored and analyzed using R version 4.0.5 [16]. The analysis involved a pilot study with 35 subjects, where continuous variables such as age and PSA levels were summarized by their median and interquartile ranges, while categorical factors like Gleason score, pathological T stage, and polymorphisms were summarized through counts and percentages. Hardy-Weinberg equilibrium was assessed, and Odds Ratios (OR) were calculated for standard genomic models. The pilot study data informed sample size calculations for three genetic models: dominant, recessive, and additive, assuming that the test model was either dominant or recessive. Data from the full study were summarized similarly. ORs were computed for general, dominant, recessive, multiplicative, and additive models, with the null hypothesis of OR = 1 tested for each model. Specific ORs were calculated for subsets of cases based on PSA levels (PSA < 10 vs. PSA ≥ 10), Gleason scores (≤ 7 vs. > 7), and pathological stages (pT1 and pT2 vs. pT3 and pT4).

Gene expression data analysis was described previously [15]. Briefly, normality of the fold change (FC) was assessed by the quantile-quantile plot (qqPlot) with the 95% confidence band constructed by bootstrap. The logarithmic transformation (with base 2) of FC was used and normality of the log-transformed FC was assessed by qqPlot. Log-transformed FC data were explored using boxplots, overlaid with swarmplot of the data points. The null hypothesis of the equality of the population means of log-FC in the two subpopulations was tested by the two-sample two-sided t test. Findings with p-values below 0.05 were considered statistically significant.

## Results

The allele and genotype frequencies for the *CDK4* rs2069502, *CCND1* rs9344, *p16*^*INK4a*^ rs11515, and *RB* rs3092904 polymorphisms are summarized in Table 2. Among controls, genotype frequencies did not deviate significantly from Hardy-Weinberg equilibrium (*p*>0.05), except for the *CDK6* rs2285332 and *p15*^*INK4b*^ rs3217986 polymorphisms, which exhibited significant deviations (*p*<0.05) and were therefore excluded from further analysis. We observed a significant association between the *GA* genotype of the *CCND1* rs9344 polymorphism and an increased risk of prostate cancer (OR, 1.64; 95% CI, 1.23–2.20; *p* < 0.001). However, no significant associations were found between the *CDK4* rs2069502, *p16*^*INK4a*^ rs11515, or *RB* rs3092904 polymorphisms and prostate cancer risk.

**Table 2:** Genotype, allele distribution and association analysis of *CDK4* rs2069502, *CCND1* rs9344, *p16*^*INK4a*^ rs11515, *RB* rs3092904 polymorphisms and risk of prostate cancer

Genotype	Healthy controls, *n*	Prostate cancer, *n*	OR (95%CI)	*p*-Value
***CDK4 rs2069502***				
*CC*	236	191	1.00 (ref.)	
*CT*	231	206	1.10 (0.84–1.44)	0.50
*TT*	64	71	1.37 (0.93–2.02)	0.11
Allele				
*C*	703	588	1.00 (ref.)	
*T*	359	348	1.16 (0.96–1.39)	0.12
***CCND1*** **rs9344**				
*GG*	161	115	1.00 (ref.)	
*GA*	274	322	**1.64 (1.23–2.20)**	**0.001**
*AA*	132	95	1.01 (0.71–1.44)	1.00
Allele				
*G*	596	552	1.00 (ref.)	
*A*	538		1.03 (0.87–1.22)	0.77
***p16***^***INK4a***^ **rs11515**				
*GG*	406	373	1.00 (ref.)	
*GC*	149	137	1.00 (0.76–1.31)	1.00
*CC*	9	17	2.04 (0.91–4.88)	0.11
Allele				
*G*	961	883	1.00 (ref.)	
*C*	167	171	1.11 (0.88–1.41)	0.38
***RB*** **rs3092904**				
*TT*	279	268	1.00 (ref.)	
*TA*	238	216	0.95 (0.74–1.21)	0.66
*AA*	50	45	0.94 (0.60–1.45)	0.84
Allele				
*T*	796	752	1.00 (ref.)	
*A*	338	306	0.96 (0.80–1.15)	0.67

Further stratification of the cases based on clinicopathological characteristics such as serum PSA levels (< 10 and ≥ 10 ng/ml), Gleason score (≤ 7 and > 7), and pathological T stage (pT1/pT2 and pT3/pT4) is provided in Table 3. No significant associations were observed between the serum PSA levels, Gleason score, or pathological T-stage and the *CDK4* rs2069502, *CCND1* rs9344, *p16*^*INK4a*^ rs11515 and *RB* rs3092904 polymorphisms.

**Table 3:** Association of the *CDK4* rs2069502, *CCND1* rs9344, *p16*^*INK4a*^ rs11515, *RB* rs3092904 genotypes with clinicopathological features of prostate cancer

Genotype	PSA < 10 ng/ml, vs. PSA ≥ 10 ng/ml		Gleason score ≤ 7 vs. Gleason score >7		pT1/pT2 vs. pT3/pT4	
	OR (95%CI)	*p*-Value	OR (95%CI)	*p*-Value	OR (95%CI)	*p*-Value
***CDK4 rs2069502***						
*CC*	1.00 (ref.)		1.00 (ref.)		1.00 (ref.)	
*CT*	0.86 (0.55–1.33)	0.51	1.01 (0.64–1.61)	1.00	1.29 (0.73–2.29)	0.39
*TT*	1.41 (0.76–2.66)	0.28	1.00 (0.52–1.89)	1.00	1.28 (0.54–3.10)	0.66
***CCND1*** **rs9344**						
*GG*	1.00 (ref.)		1.00 (ref.)		1.00 (ref.)	
*GA*	1.04 (0.65–1.66)	0.91	1.32 (0.80-2-22)	0.31	0.65 (0.32–1.30)	0.30
*AA*	0.81 (0.44–1.49)	0.54	1.04 (0.53-2.00)	1.00	0.55 (0.21–1.43)	0.24
***p16***^***INK4a***^ **rs11515**						
*GG*	1.00 (ref.)		1.00 (ref.)		1.00 (ref.)	
*GC*	0.75 (0.49–1.15)	0.19	1.15 (0.73–1.82)	0.56	0.95 (0.52–1.71)	0.88
*CC*	1.55 (0.50–5.37)	0.57	0.41 (0.06–1.61)	0.35	0.37 (0.01–3.25)	0.62
***RB*** **rs3092904**						
*TT*	1.00 (ref.)		1.00 (ref.)		1.00 (ref.)	
*TA*	1.26 (0.84–1.88)	0.26	1.04 (0.68–1.58)	0.91	1.48 (0.85–2.59)	0.21
*AA*	0.78 (0.37–1.60)	0.59	1.25 (0.60–2.50)	0.59	0.57 (0.20–1.48)	0.34

The relative mRNA expression levels of *CDK4*,* CDK6*,* CCND1*,* p16*^*INK4a*^, *p15*^*INK4b*^, and *RB* were assessed in 44 prostate cancer tissues and 31 BPH tissues using qRT-PCR. The relative mRNA expression of *CDK4*, *CDK6*, and *p16*^*INK4a*^ in prostate tumor tissues did not differ significantly from that in BPH tissues [*CDK4*: median log_2_(FC) value: 0.02, *p* = 0.77; *CDK6*: median log_2_(FC) value: -0.44, *p* = 0.09; and *p16*^*INK4a*^: median log_2_(FC) value: 0.49, *p* = 0.91]. The relative mRNA expression of *CCND1*, *p15*^*INK4b*^ and *RB* was significantly lower in prostate tumor tissues compared to BPH tissues [*CCND1*: median log_2_(FC) value: -2.12, *p* = 1 × 10^− 12^; *p15*^*INK4b*^: median log_2_(FC) value: -0.85, *p* = 0.002; *RB*: median log_2_(FC) value: -1.28; *p* = 0.002].

Consequently, we evaluated the effect of the *CDK4* rs2069502, *CCND1* rs9344, *p16*^*INK4a*^ rs11515, and *RB* rs3092904 polymorphisms on the relative mRNA expression levels of their corresponding genes. No significant effect was observed for the *CDK4* rs2069502 *CT* and *TT* mutant genotypes on *CDK4* relative mRNA expression compared to the *CC* genotype [median log_2_(FC) value: 0.06, -0.17 and − 0.07; *p* = 0.76]. For the *CCND1* rs9344 polymorphism, no significant change in *CCND1* mRNA expression was found in patients with *GA* and *AA* genotypes compared to those with the *GG* genotype [median log_2_(FC) value: -1.29; -1.25 and − 1.88; *p* = 0.55]. Analysis of the *p16*^*INK4a*^ rs11515 polymorphism revealed that patients with the *GC* genotype had lower relative expression of *p16*^*INK4a*^ mRNA compared to those with the *GG* genotype [median log_2_(FC): -0.23 and 0.40; *p* = 0.47]. The homozygous mutant *CC* genotype of *p16*^*INK4a*^ rs11515 was absent from the study population.

**Fig. 1:**
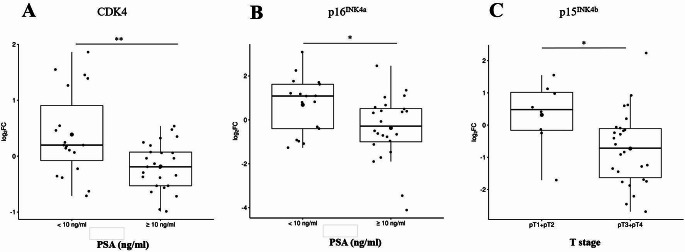
**A** Box plot showing the correlation between the relative *CDK4* mRNA expression [represented as log_2_ fold change (FC) values] and serum PSA levels. **B** Box plot showing the correlation between the relative *p16*^*INK4a*^ mRNA expression [represented as log_2_ fold change (FC) values] and serum PSA levels. **C** Box plot showing the correlation between the relative *p15*^*INK4b*^ mRNA expression [represented as log_2_ fold change (FC) values] and pathological T stage. Box plots represent medians and interquartile ranges of relative mRNA expression. Whiskers represent minimum and maximum 1.5 interquartile range and dots are outliers. (*P < 0.05; **P < 0.01)

We further investigated the association between changes in the relative mRNA expression of *CDK4*, *CDK6*, *CCND1*, *p16*^*INK4a*^, *p15*^*INK4b*^, *RB* and clinicopathological features (serum PSA values, Gleason score, and pathological T stage). In patients with PSA levels ≥10 ng/ml, *CDK4* mRNA expression was significantly reduced compared to patients with PSA < 10 ng/ml [median log_2_(FC) value: -0.19 and 0.19; *p* = 0.007; Fig. 1A]. Similarly, a significant reduction in relative *p16*^*INK4a*^ mRNA expression was observed in patients with PSA ≥ 10 ng/ml compared to those with PSA < 10 ng/ml [median log_2_(FC) value: -0.29 and 1.09; *p* = 0.02; Fig. 1B]. Furthermore, patients with pathological T stage pT3/pT4 exhibited significantly lower relative expression of *p15*^*INK4b*^ mRNA compared to those with pT1/pT2 [median log_2_(FC) value: -0.72 and 0.48; *p* = 0.03; Fig. 1C]. No significant effects were observed for the remaining clinicopathological features on the relative *CDK4*, *CDK6*, *CCND1*, *p16*^*INK4a*^, *p15*^*INK4b*^, or *RB* mRNA expression.

## Discussion

Androgens are well-established drivers of prostate cancer progression. The androgen receptor (AR)-androgens axis induces cell cycle progression, partly through the upregulation of cyclin D1 expression and the subsequent activation of CDK4/6. The activated CDK4/6 complex phosphorylates the RB protein, facilitating the cell´s progression through the cell cycle. Dysregulation of this pathway – via cyclin D overexpression, CDK4/6 amplification or mutation, or the loss of CDK4/6 inhibitors or RB – can result in unchecked tumor cell proliferation [6, 17, 18]. To the best of our knowledge, this is the first study to delineate the association between *CDK4* rs2069502, *p16*^*INK4a*^ rs11515, *RB* rs3092904 polymorphisms and prostate cancer risk, as well as their influence on corresponding mRNA levels. In this Slovak case-control study, we identified an association between the *GA* genotype of the *CCND1* rs9344 polymorphism and an increased risk of prostate cancer. Additionally, we observed significantly reduced mRNA expression levels of *CCND1*, *p15*^*INK4b*^, and *RB* in prostate tumor tissues compared to BPH tissues, while the expression levels of *CDK4*, *CDK6*, and *p16*^*INK4a*^ remained unaltered. Moreover, tumors with lower relative *CDK4* and *p16*^*INK4a*^ mRNA expression levels were associated with elevated serum PSA levels, and lower relative expression of *p15*^*INK4b*^ was linked to higher pathological T stages (pT3/pT4).

The role of the *CDK4* rs2069502 polymorphism in human prostate carcinogenesis has not been previously investigated, though it has been examined in breast cancer [19, 20]. These studies did not identify a significant association between the *CDK4* rs2069502 polymorphism and breast cancer risk, a finding consistent with our results for prostate cancer. Studies on other malignancies, including adrenocortical carcinoma [21], osteosarcoma [18], lung cancer [22], and nasopharyngeal carcinoma [23], have demonstrated overexpression of CDK4 and CDK6, which promote tumor progression and poor prognosis. In prostate cancer, the roles of CDK4 and CDK6 remain less clear. Halvorsen et al. [24] reported no significant differences in CDK4 protein expression between localized prostate cancer and BPH tissues, suggesting no apparent alteration in CDK4 protein levels. However, Chen et al. [25] found higher CDK6 expression in prostate tumors compared to normal tissues and demonstrated that three CDK6 inhibitors (apigenin, chrysin, and fisetin) could reduce CDK6 expression and inhibit cell proliferation. Beyond its established role in G_1_ cell cycle progression, CDK6 has been implicated in the stimulation of AR activity, potentially contributing to prostate cancer development and progression [26]. Contrary to these findings, our study revealed no significant differences in relative *CDK4* and *CDK6* mRNA expression levels between prostate tumor and BPH tissues. Interestingly, we observed a statistically significant decrease in relative *CDK4* mRNA expression in patients with serum PSA levels ≥ 10 ng/ml. This suggests that additional molecular mechanisms may lead to the dysfunction of the cyclin D-CDK4/6-RB pathway in prostate cancer, ultimately resulting in the same biological consequence: progression through the G_1_/S phase of the cell cycle.

The common *CCND1* rs9344 (G870A) polymorphism at codon 242 influences splicing at the exon 4–intron 4 boundary, resulting in an alternative transcript, CCND1b, which lacks exon 5 [27]. CCND1b is thought to function as a nuclear oncogene, independent of the canonical CCND1a transcript [28]. Several studies and one meta-analysis have reported the role of the *CCND1* rs9344 polymorphism and the risk of prostate cancer [11–13, 29]. In our study, we observed a significant association between the *GA* genotype of the *CCND1* rs9344 polymorphism and an increased risk of prostate cancer. Few studies have reported that the *AA* genotype is associated with an increased risk of prostate cancer [12, 13], whereas other study reported no effect of this polymorphism on prostate cancer risk [29]. A meta-analysis conducted by Zheng et al. [11], which included 3,820 cases and 3,825 controls, concluded that the *CCND1* rs9344 polymorphism was not significantly associated with prostate cancer risk. The discrepancies among studies may stem from differences in patient populations, including sample size, ethnic diversity, and genetic heterogeneity. These factors highlight the importance of considering population-specific genetic and environmental influences when interpreting the role of the *CCND1* rs9344 polymorphism in prostate cancer susceptibility.

Some studies have focused on the prognostic value of cytoplasmic and/or nuclear CCND1 expression in prostate cancer, with the majority focusing on its nuclear localization [30, 31]. Cao et al. [30] reported that cytoplasmic CCND1 expression was significantly lower in tumor tissues compared to adjacent normal tissues. Furthermore, their findings indicated that the combined evaluation of cytoplasmic and nuclear CCND1 expression did not provide a better prognostic value than cytoplasmic CCND1 alone. Study of Comstock et al. [32] demonstrated that cytoplasmic localization of CCND1 was predominant in low-grade prostate carcinomas, whereas nuclear CCND1 was associated with high-grade tumors. Considering all the data cited, it has been proposed that, although the total expression of CCND1 is more abundant in normal tissues and concentrated in the cytoplasm, CCND1 in tumor cells is more likely to be transported and accumulated in the nucleus [30]. From our analysis, we observed a significantly decreased relative *CCND1* mRNA expression in prostate tumor tissues compared to BPH tissues. We hypothesize that changes in the expression of individual genes within a specific molecular pathway may be regulated by mechanisms associated with microRNA (miR) activity [33]. Previous study has demonstrated that overexpressed miR-15a and miR-16-1 can directly bind to the 3’-untranslated region (3’-UTR) of *CCND1*, thereby reducing its expression levels [34]. Further research is warranted to clarify this mechanism and to identify specific target molecules that influence *CCND1* gene expression.

The association of the *p16*^*INK4a*^ rs11515 polymorphism, located in 3’-UTR of exon 3, has been extensively studied in various tumors [35, 36], but not in prostate cancer. However, these studies have reported contradictory findings regarding which allele is associated with an increased risk for cancer and plays a more critical role. This indicates that polymorphisms within the 3’-UTR of the *p16*^*INK4a*^ gene may influence cancer phenotypes and disease susceptibility by modifying microR regulation [37]. It has been demonstrated that the *p16*^*INK4a*^ rs11515 polymorphism is predicted to affect the miR-601 binding and that the G500 allele is associated with increased *p16*^*INK4a*^ expression [38]. Two discrete tumor-associated states for p16^INK4a^ have been reported: loss or silencing, and elevated protein expression related to RB loss of function [39]. P16^INK4a^ protein expression in prostate cancer is heterogeneous, which could be attributed to altered *p16*^*INK4a*^ mRNA expression or potentially to changes in translation and/or protein stability [24]. Some studies have identified overexpression of *p16*^*INK4a*^ mRNA in prostate tumors compared to the adjacent normal prostate or BPH tissues [40, 41]. In our study, we observed similar relative *p16*^*INK4a*^ mRNA expression levels in prostate tumor tissues and BPH tissues, along with a significantly decreased relative expression of *p16*^*INK4a*^ mRNA in patients with serum PSA levels ≥ 10 ng/ml.

In human cells, the inactivation of p15^INK4b^ facilitates cell cycle progression. P15^INK4b^ is often regarded as functionally equivalent to p16^INK4a^. However, Xia et al. [42] demonstrated that p15^INK4b^ is markedly stronger than p16^INK4a^ in inhibiting RB phosphorylation, E2F activity, and cell cycle progression. Previous study has shown that p15^INK4b^ expression levels are relatively uniform between tumors and normal matched prostates [40]. A subsequent study reported that both p15^INK4b^ and p16^INK4a^ are expressed more frequently in prostate carcinomas compared to benign prostatic tissues [43]. In the present study, the *p15*^*INK4b*^ mRNA expression level was significantly lower in prostate tumor tissues than in BPH tissues. Moreover, we found that lower relative *p15*^*INK4b*^ mRNA expression was associated with a higher pathological T stage (pT3/pT4). Several potential mechanisms could be proposed related to discrepancies among the studies. These include homozygous deletions at the p15^INK4b^ locus [44], mutations in the *p15*^*INK4b*^ gene [45], or transcriptional repression by DNA methylation of the CpG island in the 5’ region of *p15*^*INK4b*^ [44]. Such epigenetic and genetic alterations have been documented in various tumors, suggesting that abnormalities in p15^INK4b^ may be tumor-specific.

Though various reports have addressed the role of the *RB* rs3092904 polymorphism in different cancers [46, 47], no study has investigated its association with prostate cancer. The present study demonstrates that *RB* rs3092904 polymorphism is not associated with the prostate cancer risk among Slovak men. Furthermore, we show that the relative *RB* mRNA expression is significantly decreased in prostate tumor tissues compared to BPH tissues. Homozygous deletion of *RB* locus and loss of RB function facilitate the development of a castrate-resistant prostate cancer via E2F-mediated upregulation of the AR [17]. A study by Thangavel et al. [48] showed that loss of RB function alters cytoskeletal organization, induces epithelial-mesenchymal transition, and increases migration, invasion, and metastasis. Additionally, RB loss modulates cancer cell sensitivity to different chemotherapeutic agens, with both elevated and diminished sensitivity reported [49]. Functionally active RB is also necessary for the effectiveness of CDK4/6 inhibitors [50].

## Conclusions

We demonstrated an association between the *CCND1* rs9344 *GA* genotype and prostate cancer risk in the Slovak population. Additionally, we showed a significant reduction in the relative mRNA expression levels of *CCND1*,* p15*^*INK4b*^ and *RB* in prostate tumor tissues compared to BPH tissues. Interestingly, we found the associations between *CDK4* and *p16*^*INK4a*^ mRNA expression with serum PSA levels, as well as between *p15*^*INK4b*^ mRNA expression and a higher pathological T stage (pT3/pT4). However, the correlation between the selected polymorphisms and their influence on corresponding mRNA levels was not confirmed. We assume that genetic variations, including polymorphisms and/or altered expression, could affect the activity of cyclin D1-CDK4 complex. In combination with the altered ability of p16^INK4b^ protein to bind to this complex, these factors may ultimately contribute to cell cycle deregulation and the development of prostate cancer. Future studies involving large, multiethnic populations are required to validate and further explore the clinical potential of these findings on a global scale.

## Data Availability

The datasets used and/or analysed during the current study are available from the corresponding author upon reasonable request.

## Abbreviations

AR: Androgen receptor
BPH: Benign prostatic hyperplasia
CI: Confidence interval
CCND1: Cyclin D1
CDK4: Cyclin dependent kinase 4
CDK6: Cyclin dependent kinase 6
miR: MicroRNA
OR: Odds ratio
PSA: Prostatic specific antigen
pT: Pathological T state
RB: Retinoblastoma protein
qRT-PCR: Quantitative real-time PCR

## References

[1] Schatten H (2018) Brief Overview of Prostate Cancer Statistics, Grading, Diagnosis and Treatment Strategies. Adv Exp Med Biol 1095:1–14. 10.1007/978-3-319-95693-0_1. PMID: 30229546 10.1007/978-3-319-95693-0_1 30229546

[2] Rebbeck TR (2017) Prostate Cancer genetics: variation by race, ethnicity, and geography. Semin Radiat Oncol 27(1):3–10. 10.1016/j.semradonc.2016.08.002 27986209 10.1016/j.semradonc.2016.08.002 PMC5175208

[3] Bergengren O, Pekala KR, Matsoukas K, Fainberg J, Mungovan SF, Bratt O, Bray F, Brawley O, Luckenbaugh AN, Mucci L, Morgan TM, Carlsson SV (2023) 2022 Update on prostate Cancer epidemiology and risk Factors-A systematic review. Eur Urol 84(2):191–206. 10.1016/j.eururo.2023.04.021 37202314 10.1016/j.eururo.2023.04.021 PMC10851915

[4] Evans AJ (2018) Treatment effects in prostate cancer. Mod Pathol 31(S1):S110–121. 10.1038/modpathol.2017.158 29297495 10.1038/modpathol.2017.158

[5] Williams GH, Stoeber K (2012) The cell cycle and cancer. J Pathol 226(2):352–364. 10.1002/path.3022 21990031 10.1002/path.3022

[6] Goel S, Bergholz JS, Zhao JJ (2022) Targeting CDK4 and CDK6 in cancer. Nat Rev Cancer 22(6):356–372. 10.1038/s41568-022-00456-3 35304604 10.1038/s41568-022-00456-3 PMC9149100

[7] Fassl A, Geng Y, Sicinski P (2022) CDK4 and CDK6 kinases: from basic science to cancer therapy. Science 375(6577):eabc1495. 10.1126/science.abc1495 35025636 10.1126/science.abc1495 PMC9048628

[8] Hamilton E, Infante JR (2016) Targeting CDK4/6 in patients with cancer. Cancer Treat Rev 45:129–138. 10.1016/j.ctrv.2016.03.002 27017286 10.1016/j.ctrv.2016.03.002

[9] Engeland K (2022) Cell cycle regulation: p53-p21-RB signaling. Cell Death Differ 29(5):946–960. 10.1038/s41418-022-00988-z 35361964 10.1038/s41418-022-00988-z PMC9090780

[10] Almalki SG (2023) The pathophysiology of the cell cycle in cancer and treatment strategies using various cell cycle checkpoint inhibitors. Pathol Res Pract 251:154854. 10.1016/j.prp.2023.154854 37864989 10.1016/j.prp.2023.154854

[11] Zheng M, Wan L, He X, Qi X, Liu F, Zhang DH (2015) Effect of the CCND1 A870G polymorphism on prostate cancer risk: a meta-analysis of 3,820 cases and 3,825 controls. World J Surg Oncol 13:55. 10.1186/s12957-015-0479-8 25888980 10.1186/s12957-015-0479-8 PMC4344796

[12] Mandal RK, Mittal RD (2012) Are cell cycle and apoptosis genes associated with prostate cancer risk in North. Indian Population?? Urol Oncol 30(5):555–561. 10.1016/j.urolonc.2010.05.006 20822933 10.1016/j.urolonc.2010.05.006

[13] Wang L, Habuchi T, Mitsumori K, Li Z, Kamoto T, Kinoshita H, Tsuchiya N, Sato K, Ohyama C, Nakamura A, Ogawa O, Kato T (2003) Increased risk of prostate cancer associated with AA genotype of Cyclin D1 gene A870G polymorphism. Int J Cancer 103(1):116–120. 10.1002/ijc.10793 12455063 10.1002/ijc.10793

[14] Vilčková M, Škereňová M, Dobrota D, Kaplán P, Jurečeková J, Kliment J, Híveš M, Dušenka R, Evin D, Knoško Brožová M, Kmeťová Sivoňová M (2023) Polymorphisms in the gene encoding CYP1A2 influence prostate cancer risk and progression. Oncol Lett 25(2):85. 10.3892/ol.2023.13671 36760517 10.3892/ol.2023.13671 PMC9878356

[15] Híveš M, Jurečeková J, Kliment J, Grendár M, Kaplán P, Dušenka R, Evin D, Vilčková M, Holečková KH, Sivoňová MK (2022) Role of genetic variations in CDK2, CCNE1 and p27KIP1 in prostate Cancer. Cancer Genomics Proteom 19(3):362–371. 10.21873/cgp.20326 10.21873/cgp.20326 PMC9016479 35430569

[16] R Core Team (2021) R: A language and environment for statistical computing. R Foundation for Statistical Computing, Vienna, Austria. URL https://www.R-project.org/

[17] Sharma A, Yeow WS, Ertel A, Coleman I, Clegg N, Thangavel C, Morrissey C, Zhang X, Comstock CE, Witkiewicz AK, Gomella L, Knudsen ES, Nelson PS, Knudsen KE (2010) The retinoblastoma tumor suppressor controls androgen signaling and human prostate cancer progression. J Clin Invest 120(12):4478–4492. 10.1172/JCI44239 21099110 10.1172/JCI44239 PMC2993601

[18] Zhou Y, Shen JK, Yu Z, Hornicek FJ, Kan Q, Duan Z (2018) Expression and therapeutic implications of cyclin-dependent kinase 4 (CDK4) in osteosarcoma. Biochim Biophys Acta Mol Basis Dis 1864(5PtA):1573–1582. 10.1016/j.bbadis.2018.02.004 29452249 10.1016/j.bbadis.2018.02.004

[19] Al-owaidi DS, Algazally ME, Alawaad AS (2020) Association of rs2069502 cyclin-dependent kinase 4 gene with breast cancer. Euromediterranean Biomedical J 15(09):40–46

[20] Driver KE, Song H, Lesueur F, Ahmed S, Barbosa-Morais NL, Tyrer JP, Ponder BA, Easton DF, Pharoah PD, Dunning AM, Studies in Epidemiology and Risks of Cancer Heredity (SEARCH) Team (2008) Association of single-nucleotide polymorphisms in the cell cycle genes with breast cancer in the British population. Carcinogenesis 29(2):333–341. 10.1093/carcin/bgm284 18174243 10.1093/carcin/bgm284 PMC2346546

[21] Lippert J, Appenzeller S, Liang R, Sbiera S, Kircher S, Altieri B, Nanda I, Weigand I, Gehrig A, Steinhauer S, Riemens RJM, Rosenwald A, Müller CR, Kroiss M, Rost S, Fassnacht M, Ronchi CL (2018) Targeted molecular analysis in adrenocortical carcinomas: A strategy toward improved personalized prognostication. J Clin Endocrinol Metab 103(12):4511–4523. 10.1210/jc.2018-01348 30113656 10.1210/jc.2018-01348

[22] Wu A, Wu B, Guo J, Luo W, Wu D, Yang H, Zhen Y, Yu X, Wang H, Zhou Y, Liu Z, Fang W, Yang Z (2011) Elevated expression of CDK4 in lung cancer. J Transl Med 9:38. 10.1186/1479-5876-9-38 21477379 10.1186/1479-5876-9-38 PMC3094221

[23] Fang W, Li X, Jiang Q, Liu Z, Yang H, Wang S, Xie S, Liu Q, Liu T, Huang J, Xie W, Li Z, Zhao Y, Wang E, Marincola FM, Yao K (2008) Transcriptional patterns, biomarkers and pathways characterizing nasopharyngeal carcinoma of Southern China. J Transl Med. 2008, 6:32. 10.1186/1479-5876-6-32 10.1186/1479-5876-6-32 PMC2443113 18570662

[24] Halvorsen OJ, Høstmark J, Haukaas S, Høisaeter PA, Akslen LA (2000) Prognostic significance of p16 and CDK4 proteins in localized prostate carcinoma. Cancer 88(2):416–424 10640976

[25] Chen X, Wu Y, Wang X, Xu C, Wang L, Jian J, Wu D, Wu G (2022) CDK6 is upregulated and May be a potential therapeutic target in enzalutamide-resistant castration-resistant prostate cancer. Eur J Med Res 27(1):105. 10.1186/s40001-022-00730-y 35780240 10.1186/s40001-022-00730-y PMC9250190

[26] Lim JT, Mansukhani M, Weinstein IB (2005) Cyclin-dependent kinase 6 associates with the androgen receptor and enhances its transcriptional activity in prostate cancer cells. Proc Natl Acad Sci USA 102(14):5156–5161. 10.1073/pnas.0501203102 15790678 10.1073/pnas.0501203102 PMC556011

[27] Knudsen KE, Diehl JA, Haiman CA, Knudsen ES (2006) Cyclin D1: polymorphism, aberrant splicing and cancer risk. Oncogene 25(11):1620–1628. 10.1038/sj.onc.1209371 16550162 10.1038/sj.onc.1209371

[28] Lu F, Gladden AB, Diehl JA (2003) An alternatively spliced Cyclin D1 isoform, Cyclin D1b, is a nuclear oncogene. Cancer Res 63(21):7056–7061 14612495

[29] Koike H, Suzuki K, Satoh T, Ohtake N, Takei T, Nakata S, Yamanaka H (2003) Cyclin D1 gene polymorphism and Familial prostate cancer: the AA genotype of A870G polymorphism is associated with prostate cancer risk in men aged 70 years or older and metastatic stage. Anticancer Res 23(6D):4947–4951 14981950

[30] Cao Z, Chen X, Xu Y, Guo F, Ji J, Xu H, He J, Sun Y, Wang F (2020) Differential expression and prognostic value of cytoplasmic and nuclear Cyclin D1 in prostate Cancer. Biomed Res Int 2020:1692658. 10.1155/2020/1692658 32566661 10.1155/2020/1692658 PMC7281841

[31] Gerke TA, Martin NE, Ding Z, Nuttall EJ, Stack EC, Giovannucci E, Lis RT, Stampfer MJ, Kantoff PW, Parmigiani G, Loda M, Mucci LA (2015) Evaluating a 4-marker signature of aggressive prostate cancer using time-dependent AUC. Prostate 75(16):1926–1933. 10.1002/pros.23090 26469352 10.1002/pros.23090 PMC4831584

[32] Comstock CE, Revelo MP, Buncher CR, Knudsen KE (2007) Impact of differential Cyclin D1 expression and localisation in prostate cancer. Br J Cancer 96(6):970–979. 10.1038/sj.bjc.6603615 17375037 10.1038/sj.bjc.6603615 PMC2360090

[33] Cai CK, Zhao GY, Tian LY, Liu L, Yan K, Ma YL, Ji ZW, Li XX, Han K, Gao J, Qiu XC, Fan QY, Yang TT, Ma BA (2012) miR-15a and miR-16-1 downregulate CCND1 and induce apoptosis and cell cycle arrest in osteosarcoma. Oncol Rep 28(5):1764–1770. 10.3892/or.2012.1995 22922827 10.3892/or.2012.1995

[34] Bonci D, Coppola V, Musumeci M, Addario A, Giuffrida R, Memeo L, D’Urso L, Pagliuca A, Biffoni M, Labbaye C, Bartucci M, Muto G, Peschle C, De Maria R (2008) The miR-15a-miR-16-1 cluster controls prostate cancer by targeting multiple oncogenic activities. Nat Med 14(11):1271–1277. 10.1038/nm.1880 18931683 10.1038/nm.1880

[35] Royds JA, Pilbrow AP, Ahn A, Morrin HR, Frampton C, Russell IA, Moravec CS, Sweet WE, Tang WH, Currie MJ, Hung NA, Slatter TL (2016) The rs11515 polymorphism is more frequent and associated with aggressive breast tumors with increased ANRIL and decreased p16 (INK4a) expression. Front Oncol 5:306. 10.3389/fonc.2015.00306 26835415 10.3389/fonc.2015.00306 PMC4720739

[36] Polakova V, Pardini B, Naccarati A, Landi S, Slyskova J, Novotny J, Vodickova L, Bermejo JL, Hanova M, Smerhovsky Z, Tulupova E, Kumar R, Hemminki K, Vodicka P (2009) Genotype and haplotype analysis of cell cycle genes in sporadic colorectal cancer in the Czech Republic. Hum Mutat 30(4):661–668. 10.1002/humu.20931 19224585 10.1002/humu.20931

[37] Ryan BM, Robles AI, Harris CC (2010) Genetic variation in MicroRNA networks: the implications for cancer research. Nat Rev Cancer 10(6):389–402. 10.1038/nrc2867 20495573 10.1038/nrc2867 PMC2950312

[38] Landi D, Gemignani F, Barale R, Landi S (2008) A catalog of polymorphisms falling in microRNA-binding regions of cancer genes. DNA Cell Biol 27(1):35–43. 10.1089/dna.2007.0650 17941804 10.1089/dna.2007.0650

[39] Witkiewicz AK, Knudsen KE, Dicker AP, Knudsen ES (2011) The meaning of p16(ink4a) expression in tumors: functional significance, clinical associations and future developments. Cell Cycle 10(15):2497–2503. 10.4161/cc.10.15.16776 21775818 10.4161/cc.10.15.16776 PMC3685613

[40] Nguyen TT, Nguyen CT, Gonzales FA, Nichols PW, Yu MC, Jones PA (2000) Analysis of cyclin-dependent kinase inhibitor expression and methylation patterns in human prostate cancers. Prostate 43(3):233–242. 10.1002/(sici)1097-0045(20000515)43:3%3C233::aid-pros10%3E3.0.co;2-s 10797499 10.1002/(sici)1097-0045(20000515)43:3<233::aid-pros10>3.0.co;2-s

[41] Wong M, Bierman Y, Pettaway C, Kittles R, Mims M, Jones J, Ittmann M (2019) Comparative analysis of p16 expression among African American and European American prostate cancer patients. Prostate 79(11):1274–1283. 10.1002/pros.23833 31111520 10.1002/pros.23833 PMC6617792

[42] Xia Y, Liu Y, Yang C, Simeone DM, Sun TT, DeGraff DJ, Tang MS, Zhang Y, Wu XR (2021) Dominant role of CDKN2B/p15INK4B of 9p21.3 tumor suppressor hub in Inhibition of cell-cycle and Glycolysis. Nat Commun 12(1):2047. 10.1038/s41467-021-22327-5 33824349 10.1038/s41467-021-22327-5 PMC8024281

[43] Zhang Z, Rosen DG, Yao JL, Huang J, Liu J (2006) Expression of p14ARF, p15INK4b, p16INK4a, and DCR2 increases during prostate cancer progression. Mod Pathol 19(10):1339–1343. 10.1038/modpathol.3800655 16799475 10.1038/modpathol.3800655

[44] Le Frère-Belda MA, Cappellen D, Daher A, Gil-Diez-de-Medina S, Besse F, Abbou CC, Thiery JP, Zafrani ES, Chopin DK, Radvanyi F (2001) p15(INK4b) in bladder carcinomas: decreased expression in superficial tumours. Br J Cancer 85(10):1515–1521. 10.1054/bjoc.2001.2106 11720438 10.1054/bjoc.2001.2106 PMC2363957

[45] Yu C, Wang W (2016) Relationship between P15 gene mutation and formation and metastasis of malignant osteosarcoma. Med Sci Monit 22:656–661. 10.12659/msm.895022 26921270 10.12659/MSM.895022 PMC4772913

[46] Lesueur F, Song H, Ahmed S, Luccarini C, Jordan C, Luben R, Easton DF, Dunning AM, Pharoah PD, Ponder BA (2006) Single-nucleotide polymorphisms in the RB1 gene and association with breast cancer in the British population. Br J Cancer 94(12):1921–1926. 10.1038/sj.bjc.6603160 16685266 10.1038/sj.bjc.6603160 PMC2361346

[47] Anaya-Pava EJ, Nares-Cisneros J, Cárdenas-Hernández RI, Jaramillo-Rodríguez Y, Zambrano-Galván G (2017) Study of polymorphisms in the *TP53* and *RB1* genes in children with retinoblastoma in Northern Mexico. Mol Vis 23:20–25 28210099 PMC5287443

[48] Thangavel C, Boopathi E, Liu Y, Haber A, Ertel A, Bhardwaj A, Addya S, Williams N, Ciment SJ, Cotzia P, Dean JL, Snook A, McNair C, Price M, Hernandez JR, Zhao SG, Birbe R, McCarthy JB, Turley EA, Pienta KJ, Feng FY, Dicker AP, Knudsen KE, Den RB (2017) RB loss promotes prostate Cancer metastasis. Cancer Res 77(4):982–995. 10.1158/0008-5472.CAN-16-1589 27923835 10.1158/0008-5472.CAN-16-1589 PMC5700768

[49] Rebucci M, Michiels C (2013) Molecular aspects of cancer cell resistance to chemotherapy. Biochem Pharmacol 85(9):1219–1226. 10.1016/j.bcp.2013.02.017 23435357 10.1016/j.bcp.2013.02.017

[50] Sharma A, Comstock CE, Knudsen ES, Cao KH, Hess-Wilson JK, Morey LM, Barrera J, Knudsen KE (2007) Retinoblastoma tumor suppressor status is a critical determinant of therapeutic response in prostate cancer cells. Cancer Res 67(13):6192–6203. 10.1158/0008-5472.CAN-06-4424 17616676 10.1158/0008-5472.CAN-06-4424 PMC4133940

